# Fungicide sensitivity of grapevine bacteria with plant growth-promoting traits and antagonistic activity as non-target microorganisms

**DOI:** 10.1007/s11274-023-03569-5

**Published:** 2023-03-17

**Authors:** Marco Andreolli, Silvia Lampis, Lorenzo Tosi, Viviana Marano, Giacomo Zapparoli

**Affiliations:** 1 Department of Biotechnology, University of Verona, Strada Le Grazie 15, Verona, 37134 Italy; 2 AGREA Centro Studi, San Giovanni Lupatoto, Italy

**Keywords:** Fungicides, Bacteria, Plant growth promotion, Antagonistic activity, Grapevine, Non-target microorganisms

## Abstract

This study evaluates the capacity of commercial formulations of synthetic fungicides to inhibit grapevine bacterial growth when sprayed on vineyards to control diseases, such as downy mildew, powdery mildew and secondary rots. Fungicide sensitivity plate assays were carried out on bacteria isolated from vineyards that were also identified and characterized for their plant growth-promoting (PGP) traits and antifungal activity. The high taxonomic variability of bacteria screened with different chemical classes of fungicides is one new finding of this study. Seven out of 11 fungicides were able to inhibit the growth of bacteria at a concentration corresponding to the maximum dose allowed by law in spray treatments of vineyards. Bacterial sensitivity to each fungicide varied greatly. Many sensitive isolates displayed PGP traits and/or antagonistic activity. This study shows the potential impact of fungicidal treatments on grapevine bacterial microbiota. The involvement of bacteria beneficial to the growth and health of plants underlines the importance of this investigation. Our data reveal that the control of a certain disease may be possible using fungicides that have no or low impact on natural non-target microbiota. Understanding the action mechanisms of the active ingredients in these products is a priority for the development of new eco-friendly pesticides.

**Supplementary Information:**

The online version contains supplementary material available at 10.1007/s11274-023-03569-5.

## Highlights

Effects of synthetic fungicides on grapevine bacteria were analyzed.

Plant growth promoting (PGP) traits and antifungal activity of strains were assayed.

Great variability was observed among bacteria regarding fungicide sensitivity.

Many sensitive strains had PGP traits and antifungal activity.

Potential impact of fungicides on grapevine bacterial microbiota.

**Supplementary Information:**

The online version contains supplementary material available at 10.1007/s11274-023-03569-5.

## Introduction

The grapevine (*Vitis vinifera*) is the most widely cultivated fruit crop in the world and has enormous economic value. The susceptibility of almost every part of this crop to diseases causes substantial economic losses in the grape and wine sectors. Fungi are the most important agents of grapevine diseases, so fungicides, mostly synthetic, are frequently used to treat this crop. Grapevine synthetic fungicides are made from different chemical classes of molecules (e.g., anilinopyrimidine, dithiocarbamate, triazole, strobilurin, carboxamide and phenylamide) and new ones are being developed by companies in a bid to improve their effectiveness in controlling crop pathogens. However, the intensive application of fungicides, together with other pesticides deployed against bacteria, insects and weeds, gives rise to serious concerns about agricultural sustainability (Sergazina et al. 2021).

The accumulation of fungicides affects the biodiversity, including that of microorganisms, in the crop field and the surrounding ecosystems (Gikas et al. 2022). In fact, fungicides can alter the composition of bacterial and fungal communities due to their effects on non-target organisms (Marinho et al. 2020). Schaeffer et al. (2017) reported that fungicides reduced fungal richness and diversity in exposed flowers, but did not affect the bacterial community. Other studies confirmed the impact of fungicides on the soil microbial community, including bacteria (Sułowicz et al. 2016; Wang et al. 2020; Zhang et al. 2021). The toxicity of grapevine fungicides with regard to the cell viability and growth of various bacteria has previously been described (Marinho et al. 2020). Moreover, the effects of fungicides on wine yeasts may directly or indirectly affect the fermentation process (Oliva et al. 2020).

The impact of fungicides on non-target microorganisms is even more important when such microorganisms are pathogen antagonists and have plant growth-promoting (PGP) traits. Ahemad and Saghir Khan (2012) reported that fungicide stress can reduce the PGP activities of a *Pseudomonas putida* strain used as bioinoculant. The screening of pesticide-tolerant non-target PGPR bacteria for use as biofertilizers has been also recommended (Shen et al. 2019). Data on the sensitivity of bacteria to fungicides are fundamental in promoting the combined use of a bacterial inoculum for biocontrol and pesticides as sustainable disease management. Moreover, fungicides can negatively affect the composition of natural bacterial communities that otherwise could be very helpful for plant development and pathogen control. However, information about this impact on non-target microbial populations, including useful ones living on crops, is still very limited.

This study investigates the sensitivity of bacteria associated with grapevines (epiphytic, endophytic and rhizospheric strains) to some of the most common synthetic commercial fungicides used to control fungal diseases in vineyards, such as downy mildew, powdery mildew and secondary rots. Plate assays were carried out to assess the inhibitory effects of fungicides on bacterial growth at a concentration corresponding to the maximum dose allowed in spray treatments for vineyards. Several different isolates, including grapevine bacteria that were isolated and identified in this study, were analyzed and characterized for their PGP traits and antagonistic activity against fungi. To the best of author’s knowledge, this is the first investigation into the evaluation of fungicide sensitivity of high numbers of epiphytic, endophytic and rhizospheric grapevine bacteria, including those beneficial to plants. The significance of the impact of fungicides on these non-target microorganisms is discussed.

## Materials and methods

### Sampling and isolation of bacteria

Plants were sampled in abandoned or cultivated vineyards with organic methods where no synthetic fungicides were used, located in different areas (Verona, Vicenza and Trento) of a limited part of north Italy (the maximum distance between two areas was about 100 km as the crow flies) (Table S1). Leaves, petioles and portions of 1-, 2-, or 3-old year steams of *Vitis vinifera* or unidentified *Vitis* spp. rootstock were collected to isolate the bacteria. The isolation of endophytic bacteria, from leaves or steams was carried out using the protocol described by Andreolli et al. (2016). Briefly, plant samples were vigorously washed in distillated water for 5 min and their surface treated for 10 min with a 1% (w/v) sodium hypochlorite (NaOCl) solution. Then, after rinsed these portions three times in sterile distilled water, small pieces were collected, using a sterile blade, in sterile 2-ml Eppendorf tubes containing 0.5-1.0 mL of physiological solution (0.9% w/v NaCl). The tubes were placed for 1 h on an orbital shaker at 27 °C, then serial dilutions were plated on R2A-agar (yeast extract 0.5 g/L, peptone 0.5 g/L, casein acid hydrolysate 0.5 g/L, glucose 0.5 g/L, starch 0.5 g/L, sodium pyruvate 0.3 g/L, K_2_HPO_4_ 0.3 g/L, MgSO_4_·7H_2_O 0.05 g/L, agar 15.0 g/L). All plates were incubated for 3–5 days at 27 °C, then colonies were picked up and purified by repeated streaking on the same medium. The absence of epiphytic microorganisms was verified by plating 200 µl of water derived from the third rinsing of plant portions used to isolate endophytic bacteria on the medium.

More than 100 isolates were considered and 58 out of them were selected (Table S1) according to the isolation sample, colony and cell morphology.

### Identification of bacteria

Identification of isolates was carried out through the sequencing of 16 S rRNA gene. Total DNA was extracted from bacterial cultures as previously described (Andreolli et al. 2011) and 16 S rRNA gene amplification conditions were described by Andreolli et al. (2016). Amplicons were purified using a commercial kit (NucleoSpin gel and PCR Clean-up, Macherey-Nagel, Düren, Germany), then the sequencing was carried out by Eurofins Genomics (Eurofins Genomics, Edersberg, Germany) in both directions using the same primers used for amplification. Sequences were searched for similarity by relying on the EzTaxon-E (www.ezbiocloud.net/resources/16s_download). The identification was gained considering the highest score obtained in each alignment provided by EzBio-Cloud’s Identify service that searches the sequence similarity against a database of 16 S rRNA sequences quality-controlled.

### Characterization of plant growth promoting traits in bacteria

The ability to solubilize phosphate by bacteria was carried out in plate assay using two media, National Botanical Research Institute’s phosphate growth medium (NBRIP) and Pikovskaya medium (PVK) (Nautiyal 1999) that were supplemented with Ca_3_(PO_4_)_2_ or CaHPO_4_. The production of siderophores by bacteria was evaluated in plate according to the protocol of Schwyn and Neilands (1987). Both assays were evaluated by measuring the clear halo size (measured along short axis) around the colony and related activity was considered as follows: 0 mm, no activity ( – ); from 1 to ≤ 2 mm, weakly positive (+/– ), from 3 to ≤ 4 mm, positive (+), > 4 mm, strongly positive (++). Assays were performed in triplicate.

#### Antagonism assay against fungi

Antagonism assay was carried out to evaluate the inhibitory activity of bacteria against fungi *Aspergillus uvarum* An3 and *Botrytis cinerea* ITEM 1719 (Lorenzini and Zapparoli 2020). A suspension of 10^6^ conidia per mL obtained from fungal culture of 7–10 days in MEA at 25 °C, were spread in Nutrient agar (Condalab, Madrid, Spain) containing 1% (w/v) malt extract and 1% (w/v) glucose. Plates were incubated at 25 °C for 24 h, then a 20 µL spot of bacterial culture grown 48–72 h in Nutrient, containing about 10^8^ cells/mL, was poured on the medium surface. Bacterial culture spots were leave to dry for 20 min in a laminar flow hood, then plates were incubated at 25 °C for 3–4 days, and examined daily. The formation of clear zone around the bacterial colony due to the lack of mycelial growth indicated antagonistic activity of bacteria against the fungus. This activity was positive (+) or strongly positive (++) when distance between margin of bacterial colony and mycelium was at least 2 and 4 mm. respectively. Assays were performed in triplicate.

### Fungicide sensitivity assay

This assay was carried out on a total of 100 bacteria, 58 isolated and identified in this study as above described and 42 isolated in previous studies (Andreolli et al. 2016; Lorenzini and Zapparoli 2020). A total of 11 commercial formulations of synthetic fungicides on sale in Italy was used (Table S2). An amount of each product (powder or concentrated emulsion), taken from the package, was diluted in water in stock solutions to use for the plate assay at desired concentration. Each fungicide was used at a concentration corresponding to the maximum dosage allowed in vineyards according to information reported in the product. Nutrient agar was used as assay medium. Final concentrations per mL of medium were: Dedalus® 2.3 µL, Lidal® 3.75 µL, Topas® 0,3 µL, Ridomil® Gold SL 0.23 µL, Cantus ® 1.20 mg, Switch® 0.8 mg, Prolectus® 50WG 1.0 mg, Tucana® 0.4 µL, Flint ® 0.25 mg, Carson® 1.35 mg, Folpan® 2.0 mg. An aliquot of diluted fungicide was poured in tube containing this medium maintained at 40 °C, then vigorously mixed and immediately poured into a sterile plate. After the solidification, plates were inoculated with 20 µL spot of bacterial culture grown 48–72 h in Nutrient, then leave to dry for 20 min in a laminar flow hood. Plates were incubated at 25 °C for 4 days, and the colony growth was examined daily. Fungicide was inhibitor when the colony diameter was shorter than 2 mm after 4 days. A plate of the same medium without fungicide was used as control. Assays were performed in triplicate.

## Results

### Identification of bacteria

A total of 14 genera was recognized by analyzing 16 S rRNA gene sequence of 58 bacteria isolated in this study, 6 from the rhizosphere and 52 from the phyllosphere (7 endophytic and 45 epiphytic) of grapevines, according to the comparative sequence analysis on web-accessible databases (Table 1). *Bacillus* and *Pseudomonas* were the most frequent genera (14 isolates each), while *Acinetobacter*, *Frigoribacterium*, *Kasakoina*, *Microbacterium*, *Micrococcus* and *Staphylococcus* were represented only by one isolate.

**Table 1:** Identification of 58 isolates isolated from grapevine by alignment of 16 S rRNA gene sequence against sequence provided by the EzTaxon-E database

Strain [Acc. Number]	Most similar type strain [Acc. Number]	Class	Similarity (%)
*Acinetobacter* sp. S1E [OP215346]	*Acinetobacter colistiniresistens* NIPH 2036(T) [KE340374]	Gammaproteobacteria	99.63
*Bacillus* sp. VAe [OP215314]	*Bacillus amyloliquefaciens* DSM 7(T) [FN597644]	Bacilli	100
	*Bacillus siamensis* KCTC 13,613(T) [AJVF01000043]		100
*Bacillus* sp. V3Be [OP215345]	*Bacillus amyloliquefaciens* DSM 7(T) [FN597644]	Bacilli	99.88
	*Bacillus siamensis* KCTC 13,613(T) [AJVF01000043]		99.88
*Bacillus* sp. V5B [OP215306]	*Bacillus amyloliquefaciens* DSM 7(T) [FN597644]	Bacilli	98.88
	*Bacillus siamensis* KCTC 13,613(T) [AJVF01000043]		98.88
*Bacillus* sp. V82 [OP215304]	*Bacillus amyloliquefaciens* DSM 7(T) [FN597644]	Bacilli	100
	*Bacillus siamensis* KCTC 13,613(T) [AJVF01000043]		100
*Bacillus* sp. V12e [OP215320]	*Bacillus zhangzhouensis* DW5-4(T) [JOTP01000061]	Bacilli	100
	*Bacillus safensis* subsp. *safensis* FO-36b(T) [ASJD01000027]		100
*Bacillus* sp. S1B [OP215344]	*Bacillus zhangzhouensis* DW5-4(T) [JOTP01000061]	Bacilli	100
	*Bacillus safensis* subsp. safensis FO-36b(T) [ASJD01000027]		100
*Bacillus* sp. S1D [OP215299]	*Bacillus zhangzhouensis* DW5-4(T) [JOTP01000061]	Bacilli	100
	*Bacillus safensis* subsp. *safensis* FO-36b(T) [ASJD01000027]		100
	*Bacillus safensis* subsp. *osmophilus* BC09(T) [KY990920]		100
*Bacillus* sp. V13C [OP215310]	*Bacillus amyloliquefaciens* DSM 7(T) [FN597644]	Bacilli	100
*Bacillus* sp. V13E [OP215308]	*Bacillus siamensis* KCTC 13,613(T) [AJVF01000043]	Bacilli	100
*Bacillus* sp. LG2 [OP215329]	*Bacillus subtilis* NCIB 3610(T) [ABQL01000001]	Bacilli	100
*Bacillus* sp. FM2 [OP215332]	*Bacillus gaemokensis* KCTC 13,318(T) [LTAQ01000012]	Bacilli	98.3
*Bacillus* sp. FM5 [OP215333]	*Bacillus siamensis* KCTC 13,613(T) [AJVF01000043]	Bacilli	99.87
*Bacillus* sp. G2 [OP215336]	*Bacillus siamensis* KCTC 13,613(T) [AJVF01000043]	Bacilli	99.88
*Bacillus* sp. G5 [OP215337]	*Bacillus wiedmannii* FSL W8-0169(T) [LOBC01000053]	Bacilli	100
	*Bacillus albus* N35-10-2(T) [MAOE01000087]		100
	*Bacillus luti* TD41(T) [MACI01000041]		100
*Curtobacterium* sp. PT2A [OP215325]	*Curtobacterium flaccumfaciens* LMG 3645(T) [AJ312209]	Actinomycetia	100
	*Curtobacterium herbarum* P 420/07(T) [AJ310413]		100
*Curtobacterium* sp. LG1 [OP215298]	*Curtobacterium flaccumfaciens* LMG 3645(T) [AJ312209]	Actinomycetia	99.62
*Curtobacterium* sp. LG5B [OP215326]	*Curtobacterium luteum* DSM 20,542(T) [X77437]	Actinomycetia	100
	*Curtobacterium oceanosedimentum* ATCC 31,317(T) [EF592577]		100
*Erwinia* sp. V81 [OP215305]	*Erwinia tasmaniensis* Et1/99(T) [CU468135]	Gammaproteobacteria	99.09
*Erwinia* sp. V13F [OP215307]	*Erwinia endophytica* BSTT30(T) [LN624761]	Gammaproteobacteria	98.99
*Frigoribacterium* sp. VO22 [OP215351]	*Frigoribacterium faeni* NBRC 103,066(T) [BJUV01000064]	Actinomycetia	99.53
*Kosakonia* sp. V7e [OP215317]	*Kosakonia cowanii* JCM 10,956(T) [BBEU01000098]	Gammaproteobacteria	99.72
*Massilia* sp. LG6 [OP215343]	*Massilia brevitalea* byr23-80(T) [EF546777]	Betaproteobacteria	98.78
*Massilia* sp. VO33 [OP215353]	*Massilia niabensis* 5420 S-26(T) [EU808006]	Betaproteobacteria	98.8
*Massilia* sp. ITAVB [OP215338]	*Massilia aurea* AP13(T) [AM231588]	Betaproteobacteria	99.87
*Massilia* sp. FM15 [OP215335]	*Massilia brevitalea* byr23-80(T) [EF546777]	Betaproteobacteria	98.79
	*Massilia jejuensis* 5317 J-18(T) [FJ969486]		98.79
*Microbacterium* sp. PT13 [OP215323]	*Microbacterium foliorum* DSM 12,966(T) [JYIU01000006]	Actinomycetia	99.76
	*Microbacterium keratanolyticum* IFO 13,309(T) [AB004717]		99.76
*Micrococcus* sp. LG3 [OP215342]	*Micrococcus luteus* NCTC 2665(T) [CP001628]	Actinomycetia	99.63
*Paenibacillus* sp. VT3 [OP215328]	*Paenibacillus nuruki* TI45-13ar(T) [KY419705]	Bacilli	100
*Pantoea* sp. V111 [OP215319]	*Pantoea brenneri* LMG 5343(T) [MIEI01000169]	Gammaproteobacteria	99.58
*Pantoea* sp. S23 [OP215347]	*Pantoea agglomerans* DSM 3493(T) [AJ233423]	Gammaproteobacteria	96.41
	*Pantoea brenneri* LMG 5343(T) [MIEI01000169]		96.4
*Pantoea* sp. V101 [OP215318]	*Pantoea septica* LMG 5345(T) [MLJJ01000077]	Gammaproteobacteria	98.83
	*Pantoea brenneri* LMG 5343(T) [MIEI01000169]		98.83
*Pantoea* sp. PT2D [OP215296]	*Pantoea herici* JZB 2,120,024(T) [KU189725]	Gammaproteobacteria	100
*Pantoea* sp. PT14 [OP215330]	*Pantoea hericii* JZB 2,120,024(T) [KU189725]	Gammaproteobacteria	99.76
*Pantoea* sp. VT2 [OP215303]	*Pantoea eucrina* LMG 2781(T) [EU216736]	Gammaproteobacteria	99.71
*Pantoea* sp. VO1 [OP215349]	*Pantoea eucrina* LMG 2781(T) [EU216736]	Gammaproteobacteria	100
*Pantoea* sp. VO21 [OP215350]	*Pantoea agglomerans* DSM 3493(T) [AJ233423]	Gammaproteobacteria	95.86
*Pantoea* sp. VO32 [OP215352]	*Pantoea ananatis* LMG 2665(T) [JMJJ01000010]	Gammaproteobacteria	97.69
*Priestia* sp. S1A [OP215302]	*Priestia aryabhattai* B8W22(T) [EF114313]	Bacilli	99.63
*Priestia* sp. S25 [OP215348]	*Priestia aryabhattai* B8W22(T) [EF114313]	Bacilli	100
*Priestia* sp. V13M [OP215311]	*Priestia aryabhattai* B8W22(T) [EF114313]	Bacilli	100
*Priestia* sp. FM1 [OP215331]	*Priestia megaterium* NBRC 15,308(T) [JJMH01000057]	Bacilli	99.88
*Priestia* sp. FM6 [OP215334]	*Priestia megaterium* NBRC 15,308(T) [JJMH01000057]	Bacilli	100
*Pseudomonas* sp. V5G [OP215316]	*Pseudomonas oryzihabitans* NBRC 102,199 (T) [BBIT01000012]	Gammaproteobacteria	99.21
	*Pseudomonas psychrotolerans* DSM 15,758(T) [FMWB01000061]		99.21
*Pseudomonas* sp. V13B [OP215309]	*Pseudomonas agarici* NCPPB 2289(T) [AKBQ01000002]	Gammaproteobacteria	99.09
	*Pseudomonas hutmensis* xwS2(T) [QJRG01000049]		99.09
*Pseudomonas* sp. VV13A [OP215300]	*Pseudomonas psychrotolerans* DSM 15,758(T) [FMWB01000061]	Gammaproteobacteria	99.16
	*Pseudomonas oryzihabitans* NBRC 102,199 (T) [BBIT01000012]		99.16
*Pseudomonas* sp. PT1e [OP215327]	*Pseudomonas lutea* DSM 17,257(T) [JRMB01000004]	Gammaproteobacteria	100
*Pseudomonas* sp. PT2e [OP215322]	*Pseudomonas graminis* DSM 11,363(T) [Y11150]	Gammaproteobacteria	99.77
*Pseudomonas* sp. VT1 [OP215297]	*Pseudomonas oryzihabitans* NBRC 102,199 (T) [BBIT01000012]	Gammaproteobacteria	100
	*Pseudomonas psychrotolerans* DSM 15,758(T) [FMWB01000061]		100
*Pseudomonas* sp. PT11 [OP215321]	*Pseudomonas lutea* DSM 17,257(T) [JRMB01000004]	Gammaproteobacteria	100
*Pseudomonas* sp. LG4M [OP215301]	*Pseudomonas syringae* KCTC 12,500(T) [KI657453]	Gammaproteobacteria	100
	*Pseudomonas congelans* DSM 14,939(T) [FNJH01000022]		100
	*Pseudomonas ficuserectae* JCM 2400(T) [AB021378]		100
*Pseudomonas* sp. LG4T [OP215312]	*Pseudomonas syringae* KCTC 12,500(T) [KI657453]	Gammaproteobacteria	100
	*Pseudomonas congelans* DSM 14,939(T) [FNJH01000022]		100
*Pseudomonas* sp. LG5A [OP215324]	*Pseudomonas syringae* KCTC 12,500(T) [KI657453]	Gammaproteobacteria	100
*Pseudomonas* sp. ITAVA [OP215313]	*Pseudomonas syringae* KCTC 12,500(T) [KI657453]	Gammaproteobacteria	100
	*Pseudomonas congelans* DSM 14,939(T) [FNJH01000022]		100
	*Pseudomonas cerasi* 58(T) [LT222319]		100
*Pseudomonas* sp. ITAVE [OP215339]	*Pseudomonas syringae* KCTC 12,500(T) [KI657453]	Gammaproteobacteria	100
	*Pseudomonas congelans* DSM 14,939(T) [FNJH01000022]		100
*Pseudomonas* sp. ITAVF [OP215340]	*Pseudomonas oryzihabitans* NBRC 102,199 (T) [BBIT01000012]	Gammaproteobacteria	99.28
	*Pseudomonas psychrotolerans* DSM 15,758(T) [FMWB01000061]		99.28
*Pseudomonas* sp. ITAVG [OP215341]	*Pseudomonas syringae* KCTC 12,500(T) [KI657453]	Gammaproteobacteria	100
	*Pseudomonas congelans* DSM 14,939(T) [FNJH01000022]		100
*Staphylococcus* sp. V3Ae [OP215315]	*Staphylococcus hominis* subsp. *novobiosepticus* GTC 1228(T) [AB233326]	Bacilli	100

### Analysis of plant growth-promoting (PGP) traits and antagonistic activity

A total of 51 out of 58 (88%) isolates exhibited PGP traits and/or antagonistic activity towards fungi. Specifically, 43 (74.1%) displayed ability to solubilize phosphate and/or produce siderophores, while 14 (24.1%) showed antifungal activity against *A. uvarum* An3 and *B. cinerea* ITEM 1719 (Table 2). Six isolates (*Bacillus* sp. V3Be, V5B and V82, *Priestia* sp. FM6 and *Pseudomonas* sp. PT1e) had at least one PGP trait and antagonistic activity. Twenty-six isolates were able to solubilize the phosphate in both forms, Ca_3_(PO_4_)_2_ and CaHPO_4_, while 9 isolates only in one form in NBRIP and/or PVK medium. *Pantoea* sp. PT2D had pronounced ability to solubilize the phosphate in both forms in the two media. Pronounced ability in either form, but only in one medium, was observed in 8 isolates (*Curtobacterium* sp. PT2A, *Priestia* sp. V13M, *Priestia* sp. FM1 and *Pseudomonas* sp. ITAVF in NBRIP medium, *Erwinia* sp. V13F, *Pantoea* sp. PT14, *Priestia* sp. FM6 and *Pseudomonas* sp. LG4M in PVK medium). Other isolates (e.g., *Bacillus* sp. V5B, *Microbacterium* sp. PT13, *Paenibacillus* sp. VT3) produced halos only on one specific combination of medium and phosphate source. The production of siderophores was exhibited by 20 isolates (51.7%) and 13 of them were also able to solubilize the phosphate. In particular, *Pseudomonas* sp. ITAVE and *Pseudomonas* sp. LG4T were positive in all PGP trait assays.

**Table 2:** Plant growth promoting (phosphate solubilization using NBRIP and PVK media supplemented with Ca_3_(PO_4_)_2_ or CaHPO_4_, and siderophores production) traits and antagonistic activity of 58 bacteria isolated from grapevines against *Aspergillus niger* An3 (An) and *Botrytis cinerea* ITEM 1719 (Bc). Activities were estimated measuring the radius of clear (phosphate solubilization and antagonism) or colored (siderophores) zone around colony as (−) negative, (+/−) weakly positive, (+) positive and (++) strongly positive

isolate	Plant Growth Promoting						Antagonism	
	NBRIP		PVK		Sider.		An	Bc
	Ca_3_(PO_4_)_2_	CaHPO_4_	Ca_3_(PO_4_)_2_	CaHPO_4_				
*Acinetobacter* sp. S1E	–	–	–	–	+/–		–	–
*Bacillus* sp. S1B	–	–	–	–	–		+	++
*Bacillus* sp. S1D	–	–	–	–	–		+	++
*Bacillus* sp. VAe	–	–	–	–	–		++	++
*Bacillus* sp. V3Be	–	–	–	–	+/–		++	++
*Bacillus* sp. V12e	–	–	–	–	–		–	++
*Bacillus* sp. V5B	–	–	–	+	–		++	++
*Bacillus* sp. V82	+	+	–	–	–		++	++
*Bacillus* sp. V13C	–	–	–	–	–		++	++
*Bacillus* sp. V13E	–	–	–	–	–		++	++
*Bacillus* sp. LG2	–	+	–	–	+		–	–
*Bacillus* sp. FM2	–	–	–	–	+		–	–
*Bacillus* sp. FM5	–	–	–	–	–		++	++
*Bacillus* sp. G2	–	–	–	–	–		++	++
*Bacillus* sp. G5	–	–	–	–	–		–	–
*Curtobacterium* sp. LG1	+	++	–	–	–		–	–
*Curtobacterium* sp. LG5B	–	–	+	+	–		–	–
*Curtobacterium* sp. PT2A	++	++	–	–	–		–	–
*Erwinia* sp. V81	–	–	–	–	–		–	–
*Erwinia* sp. V13F	–	–	++	++	+		–	–
*Frigoribacterium* sp VO22	+	+	+	–	+		–	–
*Kasakonia* sp. V7e	+	–	–	+/–	–		–	–
*Massilia* sp. VO33	–	–	–	–	–		–	–
*Massilia* sp. LG6	+	+/–	+/–	+/–	–		–	–
*Massilia* sp. ITAVB	–	–	–	–	+		–	–
*Massilia* sp. FM15	++	+	–	+	–		–	–
*Microbacterium* sp. PT13	–	+	–	–	–		–	–
*Micrococcus* sp. LG3	–	–	–	–	+/–		–	–
*Paenibacillus* sp. VT3	+	–	–	–	+/–		–	–
*Pantoea* sp. S23	++	+	+/–	–	–		–	–
*Pantoea* sp. VT2	++	–	–	++	+		–	–
*Pantoea* sp. PT14	–	–	++	++	–		–	–
*Pantoea* sp. PT2D	++	++	++	++	–		–	–
*Pantoea* sp. VO1	++	–	+	+/–	+		–	–
*Pantoea* sp. V101	–	–	–	–	+		–	–
*Pantoea* sp. V111	++	–	–	–	–		–	–
*Pantoea* sp. VO21	+	+/–	+/–	+/–	–		–	–
*Pantoea* sp. VO32	+	+	–	–	–		–	–
*Priestia* sp. S1A	–	–	–	–	–		–	–
*Priestia* sp. S25	–	–	–	–	–		–	–
*Priestia* sp. V13M	++	++	–	–	–	–	–	–
*Priestia* sp. FM1	++	++	–	–	–	–	–	–
*Priestia* sp. FM6	++	–	++	++	–		+/–	+
*Pseudomonas* sp. PT1e	–	+	–	–	+		++	++
*Pseudomonas* sp. PT2e	–	–	–	–	+		–	–
*Pseudomonas* sp. V5G	–	+	+	–	–		–	–
*Pseudomonas* sp. VV13A	–	–	+	–	+		–	–
*Pseudomonas* sp. V13B	+	+	–	–	–		–	–
*Pseudomonas* sp. VT1	+	+	++	+	–		–	–
*Pseudomonas* sp. PT11	–	+	–	–	+		–	–
*Pseudomonas* sp. LG4M	–	–	++	++	+		–	–
*Pseudomonas* sp. LG4T	+/–	+/–	++	+/–	+/–		–	–
*Pseudomonas* sp. LG5A	++	–	–	–	+		–	–
*Pseudomonas* sp. ITAVA	–	–	–	–	–		–	–
*Pseudomonas* sp. ITAVE	+	+	+	+	+		–	–
*Pseudomonas* sp. ITAVF	++	++	–	+/–	–		+	+/–
*Pseudomonas* sp. ITAVG	–	+	+/–	–	–		–	–
*Staphylococcus* sp. V3Ae	–	–	–	–	–		–	–

Most *Bacillus* sp. isolates displayed antagonistic activity against *A. uvarum* An3 and *B. cinerea* ITEM 1719 (Table 2). Apart from *Bacillus* sp. V12e, which strongly inhibited only on *B. cinerea* ITEM 1719, all isolates exhibited antagonism against both fungal strains. Among isolates with antagonistic activity, *Pseudomonas* sp. PT1e exhibited phosphate solubilization ability and siderophores production, *Bacillus* sp. V5B and *Bacillus* sp. V82, *Priestia* sp. FM6 and *Pseudomonas* sp. ITAVF displayed only phosphate solubilization, while *Bacillus* sp. V3Be only siderophores production.

### Fungicide sensitivity assay

**Fig. 1:**
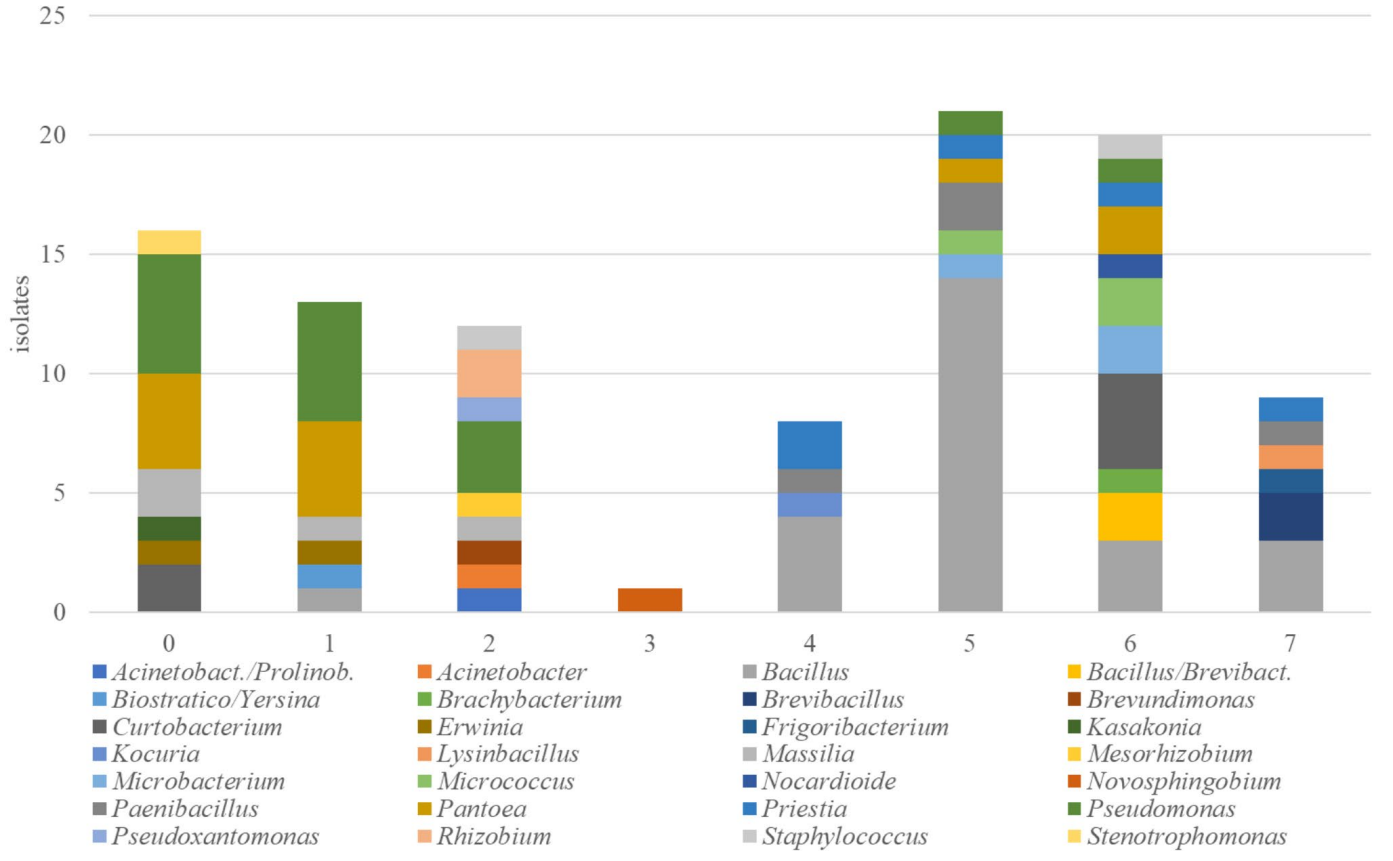
Isolate number and their related genera/OTU that are tolerant (0) or sensitive from 1 to 7 fungicides (Dedalus®, Lidal®, Topas®, Switch®, Tucana®, Carson® and Folpan®)

A total of 100 isolates, 58 isolated in this study and 42 previously investigated (Andreolli et al. 2016; Lorenzini and Zapparoli 2020), were tested for their sensitivity to 11 fungicides (Table 3). Four fungicides (Ridomil Gold®, Cantus®, Prolectus® and Flint®) did not inhibit the growth of any isolates, while the other 7 (Dedalus®, Lidal®, Topas®, Switch®, Tucana®, Carson® and Folpan®) were able to inhibit bacterial growth. A total of 16 isolates tolerated all these inhibitor fungicides, while the remaining 84 isolates were sensitive at least to one of them (Fig. 1). Twenty-six out of 28 genera/OUT had sensitive isolates. Nine isolates of *Bacillus*, *Brevibacillus*, *Frigoribacterium*, *Lysinbacillus*, *Paenibacillus* and *Priestia* were sensitive to all seven fungicides. The 16 isolates tolerant to all fungicides belonged to *Kasakonia*, *Stenotrophomonas*, *Curtobacterium*, *Erwina*, *Massilia*, *Pantoea* and *Pseudomonas*. A total of 41 isolates were sensitive to 5 or 6 fungicides. *Bacillus*, the most frequent genus in this study with 25 isolates out of 100 assayed for fungicide sensitivity, had 24 isolates sensitive at least to 4 fungicides. *Pseudomonas*, the second most represented genus with 15 isolates, had 5 tolerant and 10 sensitive isolates. Five *Pseudomonas* isolates were sensitive to only one fungicide.

**Table 3:** Results of plate assay to evaluate the sensitivity of 100 isolates (+ sensitive, – resistant) to seven commercial fungicides, Dedalus® (Ded.), Lidal® (Lid.), Topas® (Top.), Switch® (Swi.), Tucana® (Tuc.), Carson® (Car.) and Folpan® (Fol.)

isolate	Ded.	Lid.	Top.	Swi.	Tuc.	Car.	Fol.
*Acinetobacter* sp. S1E	–	–	–	–	–	+	+
*Acinetobacter*/*Prolinob*. 3Y2^1^	–	–	–	–	–	+	+
*Bacillus* sp. S1B	+	+	+	–	–	–	+
*Bacillus* sp. S1D	+	+	+	–	–	–	+
*Bacillus* sp. VAe	+	+	+	+	+	+	+
*Bacillus* sp. V3Be	+	+	+	+	+	+	+
*Bacillus* sp. V12e	+	+	+	+	–	–	+
*Bacillus* sp. V5B	+	+	+	+	–	+	+
*Bacillus* sp. V82	+	+	+	+	–	+	+
*Bacillus* sp. V13C	+	+	+	+	–	+	+
*Bacillus* sp. V13E	+	+	+	+	–	–	+
*Bacillus* sp. LG2	–	–	–	–	–	+	–
*Bacillus* sp. FM2	–	+	+	+	–	+	+
*Bacillus* sp. FM5	+	+	+	–	–	–	+
*Bacillus* sp. G2	+	+	+	+	–	–	+
*Bacillus* sp. G5	+	+	+	+	–	–	+
*Bacillus* sp. C3^2^	+	+	+	+	–	–	+
*Bacillus* sp. T9^2^	+	+	+	+	–	–	+
*Bacillus* sp. L1^2^	+	+	+	+	–	–	+
*Bacillus* sp. Pp10^2^	+	+	+	–	–	–	+
*Bacillus* sp. T2^2^	+	+	+	+	–	–	+
*Bacillus* sp. T14^2^	+	+	+	+	–	–	+
*Bacillus* sp. P3^2^	+	+	+	+	–	–	+
*Bacillus* sp. S2^2^	+	+	+	+	–	–	+
*Bacillus* sp. V20^2^	+	+	+	+	–	–	+
*Bacillus* sp. 3R1^1^	+	+	+	+	–	–	+
*Bacillus* sp. 15T31^1^	+	+	+	+	+	+	+
*Bacillus*/*Brevibact.* 15T2^1^	+	+	+	+	–	+	+
*Bacillus*/*Brevibact.* 3T15^1^	+	+	+	+	–	+	+
*Biostratico*/*Yersina* 15T3^1^	–	–	–	–	–	+	–
*Brachybacterium* sp. 3Y42^1^	+	+	+	+	–	+	+
*Brevibacillus* sp. 3Y41^1^	+	+	+	+	+	+	+
*Brevibacillus* sp. T4^2^	+	+	+	+	+	+	+
*Brevundimonas* sp. 3T21^1^	–	–	–	–	–	+	+
*Curtobacterium* sp. LG1	–	–	–	–	–	–	–
*Curtobacterium* sp. LG5B	–	–	–	–	–	–	–
*Curtobacterium* sp. PT2A	+	+	+	+	+	+	–
*Curtobacterium* sp. Pp6^2^	+	+	+	+	+	+	–
*Curtobacterium* sp. 3T2^1^	+	+	+	+	+	+	–
*Curtobacterium* sp. 15R34^1^	+	+	+	+	+	+	–
*Erwinia* sp. V81	–	–	–	–	–	–	–
*Erwinia* sp. V13F	–	–	–	–	–	+	–
*Frigoribacterium* sp VO22	+	+	+	+	+	+	+
*Kasakonia* sp. V7e	–	–	–	–	–	–	–
*Kocuria* sp. 3T22^1^	+	+	+	–	–	–	+
*Lysinbacillus* sp. 3Y22^1^	+	+	+	+	+	+	+
*Massilia* sp. VO33	–	–	–	–	–	+	+
*Massilia* sp. LG6	–	–	–	–	–	–	–
*Massilia* sp. ITAVB	–	–	–	–	–	–	+
*Massilia* sp. FM15	–	–	–	–	–	–	–
*Mesorhizobium* sp. 3Y5^1^	–	–	–	–	–	+	+
*Microbacterium* sp. PT13	+	+	+	+	–	+	+
*Microbacterium* sp. 3Y30^1^	+	+	+	+	–	+	+
*Microbacterium* sp. 15Y9^1^	+	+	+	–	–	+	+
*Micrococcus* sp. LG3	+	+	+	–	–	+	+
*Micrococcus* sp. P18^2^	+	+	+	+	–	+	+
*Micrococcus* sp. 3R6^1^	+	+	+	+	–	+	+
*Nocardioide* sp. 3Y27^1^	+	+	+	+	+	–	+
*Novosphingobium* sp. 15R31^1^	–	+	–	–	–	+	+
*Paenibacillus* sp. VT3	+	+	+	–	–	+	+
*Paenibacillus* sp. 3T16^1^	+	+	+	–	–	+	+
*Paenibacillus* sp. 3Y14^1^	+	+	+	+	+	+	+
*Paenibacillus* sp. 3Y16^1^	+	+	+	–	–	–	+
*Pantoea* sp. S23	–	–	–	–	–	+	–
*Pantoea* sp. VT2	–	–	–	–	–	–	–
*Pantoea* sp. PT14	+	+	+	+	+	+	–
*Pantoea* sp. PT2D	+	+	+	+	+	+	–
*Pantoea* sp. VO1	–	–	–	–	–	+	–
*Pantoea* sp. V101	–	–	–	–	–	–	–
*Pantoea* sp. V111	–	–	–	–	–	–	–
*Pantoea* sp. VO21	–	–	–	–	–	+	–
*Pantoea* sp. VO32	–	–	–	–	–	+	–
*Pantoea* sp. 3T6^1^	+	+	+	–	–	+	+
*Pantoea* sp. 15T1^1^	–	–	–	–	–	–	–
*Priestia* sp. S1A	+	+	+	–	–	–	+
*Priestia* sp. S25	+	+	+	–	–	+	+
*Priestia* sp. V13M	+	+	+	+	+	+	+
*Priestia* sp. FM1	+	+	+	–	–	–	+
*Priestia* sp. FM6	+	+	+	+	–	+	+
*Pseudomonas* sp. PT1e	+	+	+	–	+	–	+
*Pseudomonas* sp. PT2e	–	–	–	–	–	+	–
*Pseudomonas* sp. V5G	–	–	–	–	–	–	–
*Pseudomonas* sp. VV13A	–	–	–	–	–	–	–
*Pseudomonas* sp. V13B	–	–	–	–	–	+	+
*Pseudomonas* sp. VT1	–	–	–	–	–	–	–
*Pseudomonas* sp. PT11	–	–	–	–	–	+	–
*Pseudomonas* sp. LG4M	+	+	+	+	+	+	–
*Pseudomonas* sp. LG4T	–	–	–	–	–	+	–
*Pseudomonas* sp. LG5A	–	–	–	–	–	+	–
*Pseudomonas* sp. ITAVA	–	–	–	–	–	+	–
*Pseudomonas* sp. ITAVE	–	–	–	–	–	–	–
*Pseudomonas* sp. ITAVF	–	–	–	–	–	+	+
*Pseudomonas* sp. ITAVG	–	–	–	–	–	+	+
*Pseudomonas* sp. C19^2^	–	–	–	–	–	–	–
*Pseudoxantomonas* sp. 15R32^1^	–	+	–	–	–	+	–
*Rhizobium* sp. 3Y4^1^	–	–	–	–	–	+	+
*Rhizobium* sp. 15Y2^1^	–	–	–	–	–	+	+
*Staphylococcus* sp. V3Ae	+	+	+	+	–	+	+
*Staphylococcus* sp. Pp17^2^	–	+	–	–	–	–	+
*Stenotrophomonas* sp. 3T7^1^	–	–	–	–	–	–	–

^1^ Isolated and identified by Andreolli et al. (2016)

^2^ Isolated and identified by Lorenzini and Zapparoli (2020)

**Fig. 2:**
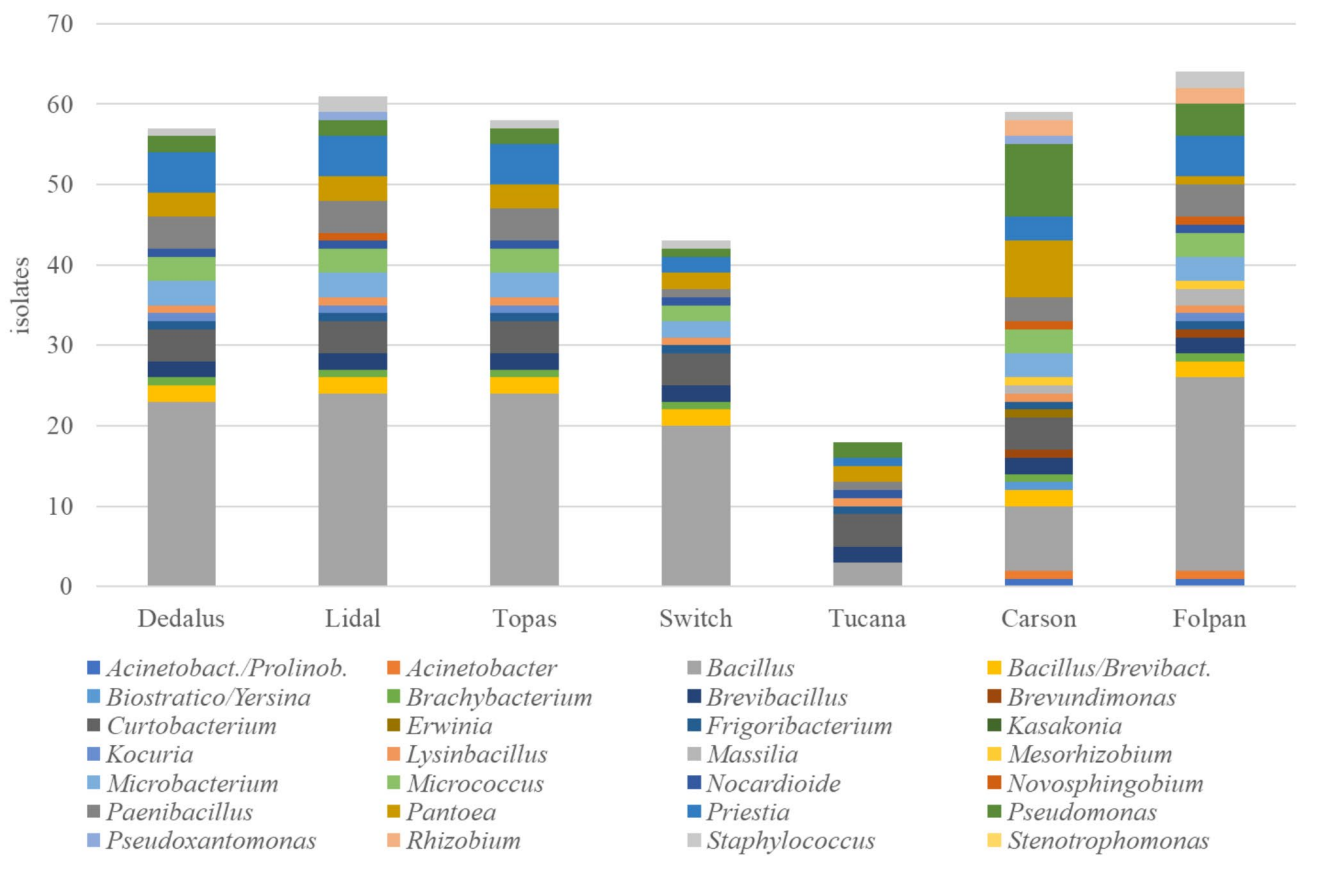
Number of isolates and their related genera/OTU sensitive to each fungicides (Dedalus®, Lidal®, Topas®, Switch®, Tucana®, Carson® and Folpan®)

Analyzing each inhibitory fungicide, Carson® affected the highest number of genera/OTU (24 out of 28) inhibiting 59 isolates, while Folpan® inhibited the highest number of isolates (64 out of 84) belonging to 22 genera/OTU (Fig. 2). Tucana® was the least inhibitory fungicide in terms of number of isolates and genera/OTU (18 and 10, respectively).

Based on the results of fungicide sensitivity of each isolate, 15 fungicide sensitive-isolate groups (S1A-S7) were recognized (Fig. S1). The most numerous group was S5A made up of by 13 isolates, all belonging to *Bacillus*, while seven groups, that is, S1B, S2B, S2C, S3, S5B, S5D and S6C, were represented only by one isolate.

Interestingly, fungicides exerted different effects on isolates with PGP traits and/or antagonistic activity, as described above. A total of 39 of 51 isolates (76%) with PGP traits and/or antagonistic activity were sensitive to fungicides. *Pseudomonas* sp. ITAVE, which had all PGP traits, was tolerant of all fungicides. Among isolates that were positive in all the phosphate splubilizing assays, *Pseudomonas* sp. VT1 and *Massilia* sp. LG6 were tolerant to 7 fungicides, while *Pantoea* sp. VO21 and *Pantoea* sp. PT2D were sensitive to 1 and 6 fungicides, respectively. It is worth mentioning that *Pseudomonas* PT1e, which is able to solubilize the phosphate in CaHPO_4_ form, produces siderophores and exerts a strong antagonistic activity, was also sensitive to 5 fungicides, *Bacillus* sp. V5B and *Bacillus* sp. V82, which displayed phosphate solubilization and antagonistic activity, were sensitive to 6 fungicides. The other *Bacillus* isolates with strong antagonistic activity (e.g., VAe, V3Be, V13C, FM5 and G2) were sensitive at least to 4 fungicides.

Most isolates (91%) with previously characterized PGP and/or antagonistic activity (Andreolli et al. 2016; Lorenzini and Zapparoli 2020) were sensitive to fungicides.

## Discussion

The isolation and identification of several bacteria associated with grapevines conducted in this study, in addition to previously analyzed isolates (Andreolli et al. 2016; Lorenzini and Zapparoli 2020), enabled these to be investigated in a representative bacterial community of this agroecosystem. Since foliar fungicides were assayed, most isolates were from the phyllosphere and only a few from the rhizosphere, although non-target impacts of active ingredients in soil microbiota have also been documented (Sułowicz et al. 2016; Roman et al. 2021).

On analyzing 58 isolates, such as *Bacillus*, *Pseudomonas*, *Pantoea* and *Curtobacterium*, most of the identified genera were frequently detected in the grapevine phyllosphere, with both endophytes and epiphytes retrieved from leaves, petioles and canes, in varying degrees of abundance (Martins et al. 2012; Andreolli et al. 2017). On the other hand, to the best of author’s knowledge, no recovery has ever been documented of *Kosakonia* from the grapevine, a genus frequently identified in other crops like rice (Walitang et al. 2017).

The high number of bacterial isolates (88%) that displayed PGP traits and/or antagonistic activity among those analyzed in the study highlights the positive potential of the microbiota for the growth and health of plants. These beneficial features of the bacteria in the phyllosphere and rhizosphere of plants, including those of the grapevine, are well documented (Etminani and Harighi 2018; Pacifico et al. 2019). Our investigation reveals great variability among bacteria with regard to phosphate solubilization and siderophore production. This diversity was particularly noticeable within isolates belonging to the same genus, such as *Pseudomonas* and *Pantoea*, represented by a high number of isolates. In addition to confirming the results of previous investigations into the phosphate solubilization ability of these bacteria (Aarab et al. 2015; Li et al. 2019), our assays, carried out in different media and forms of phosphate, suggest the involvement of different metabolisms for mineral solubilization in isolates (Alori et al. 2017). The predominance of *Bacillus* isolates among those with antagonistic activity corroborates the importance of these Firmicutes in controlling fungal pathogens (Bruisson et al. 2019). The occurrence of *Bacillus* in the grapevine phyllosphere and rhizosphere appears even more relevant considering that only another two *Pseudomonas* isolates and one *Priestia* isolate displayed such antagonistic activity. Strains with antifungal activity among these latter genera and others found in agroecosystems have been reported (Niem et al. 2020; Shahid et al. 2022). Their low incidence seen here is therefore interesting and needs to be confirmed by further investigation.

In vitro fungicide assays displayed different bacterial behaviour, including among those with PGP traits and antagonistic activity. The high number of bacterial genera/OTU screened for sensitivity to fungicides of different chemical classes is a new finding of this study. Bacteria, mainly Firmicutes such as *Bacillus*, were particularly sensitive to Folpan®, the fungicide that affected the highest number of isolates. Its active principle (folpet) is a multi-site inhibitor acting on several target sites in fungi simultaneously. Folpet and its degradation product (thiophosgene) can interact with thiols such as glutathione, an essential molecule for keeping the redox homeostasis and iron metabolism (Toledano et al. 2007; Canal-Raffin et al. 2008). Anjum et al. 2011) reported sensitivity in 35 non-identified bacteria to folpet, where the MIC was up to 1600 µg/mL. This concentration, which corresponds to the maximum dose allowed per spray application in vineyards, was used in our study to identify the sensitivity of bacteria to folpen.

Carson® (cymoxanil), used primarily to control downy mildew, affected the growth of more than 50% of assayed isolates. Cymoxanil was discovered in 1972 but its primary mechanism of action on fungi is still unknown (Hillebrand et al. 2019). In this study, the assessment of the inhibitory effects of this molecule on bacterial growth is a significant advance, given that very little information is available in the literature. Previously, Marinho et al. (2020) did not find any sensitivity of *Escherichia coli*, *Pseudomonas putida* and *Arthrobacter* sp. to 46 mg/L cymoxanil, a concentration 13 times lower than the maximum dose allowed in vineyards.

Dedalus® (tebuconazole), Lidal® (tetraconazole) and Topas® (penconazole), used to control powdery mildew in vineyards, are 1,2,4-triazole fungicides, a class of heterocyclic rings introduced in the 1970s. They act against the biosynthesis of ergosterol, an essential compound of the fungal membrane, as a mechanism of action against the target fungus (Deising et al. 2008). However, the effects of these molecules on soil bacteria has previously been documented (Zhang et al. 2014; Baćmaga et al. 2015; Sułowicz et al. 2016). Observations reported by Zhang et al. (2014), on the decrease of the ratio of gram-negative to gram-positive bacteria in soil treated with tetraconazole, are not in accordance with our study. In fact, Lidal® and the other two triazole fungicides were particularly active against gram-positive bacteria (Firmicutes were strongly inhibited, as were more than half the Actinobacteria), while they had limited effects on gram-negative bacteria (Proteobacteria). Baćmaga et al. (2015) observed changes in the relative abundance of Proteobacteria, Firmicutes, Actinobacteria and other phyla, depending on the dose of tebuconazole in the soil, and also reported the prevalence of *Bacillus*, *Brevibacillus* and *Pseudomonas* in soil treated with 10 mg/kg of this molecule. Obviously, inconsistencies between the data obtained by analyzing soil microbiota and our results are not surprising since different experimental approaches were used to evaluate the fungicide impact on bacteria. In this study, over 90% of the isolates that were sensitive to one of three molecules were also sensitive to the other two. This was expected, due to the similarity of the molecular structure of tebuconazole, tetraconazole and penconazole. Although the mechanism of their action against bacteria remains unclear, investigations into 1,2,4-triazole derivates suggest that they may have inhibitory potential against enzymatic proteins that are essential for bacteria (e.g., ATPase, DNA gyrase, glucosamie-9-phosphate synthase). These derivates bear fragments, such as quinazoline and quinazolinone, that have been demonstrated to be effective against some phytopathogen gram-negative bacteria, such as *Xanthomonas oryzae* and *Ralstonia solanacearum* (Angajala et al. 2016; Yang and Bao 2017; Shi et al. 2020). As these fungicides can induce resistance in fungal pathogenic populations in agroecosystems or other environments (Bowyer and Denning 2014), it cannot be ruled out that similar effects could occur in bacteria, an important area that requires further investigation.

The two active ingredients of Switch®, cyprodinil and fludioxonil, used to control secondary rots, target the high osmolarity glycerol pathway of fungi. In this study, this fungicide was shown to inhibit several bacteria, especially Firmicutes and Actinobacteria. According to the literature, both molecules are potentially effective against bacteria and could be effective alone or in combination. Ejim et al. (2004) observed that cyprodinil inhibited *Escherichia coli* and *Staphylococcus aureus* at concentrations more than four and two times lower than allowed in vineyards (300 mg/L), respectively. Keum et al. (2010) reported that the fludioxonil molecule acts on the pyrrolnitrin biosynthesis of bacteria and, consequently, serves as a moderate inhibitor of growth. Of course, further experiments using these two active ingredients separately must be carried out to evaluate the effects of each one on bacteria.

Tucana® (pyraclostrobin) is a strobilurin fungicide that acts on the mitochondrial respiratory chain and is mainly used to control powdery mildew in vineyards. Its effects on bacteria have previously been investigated (Skandalis et al. 2016; Lu et al. 2019). However, only Lu et al. (2019) described the in vitro inhibitory activity of this molecule in a cyanobacterium culture of *Microcystis aeruginosa*. Although Tucana® proved to be less inhibitory in terms of the number of isolates, its effects have been deployed against the genera most frequently found in agroecosystems, such as *Pseudomonas*, *Bacillus*, *Pantoea*, *Paenibacillus* and *Curtobacterium*. According to Baćmaga et al. (2015), who analyzed the effects of azoxystrobin, a strobilurin fungicide similar to pyraclostrobin, these molecules could affect the activities of enzymes like dehydrogenase, phosphatase, catalase and urease that are fundamental for bacterial growth. Our study found that Flint®, a strobilurin fungicide containing trifloxystrobin used to control powdery mildew, was ineffective in inhibiting bacteria at concentrations of 250 mg/L, which suggests that strobilurins may have different specificities on target proteins or other molecules. Further investigation to individuate their possible molecular targets should consider the high structural variability of molecules of this chemical class.

Other fungicides that did not affect the growth of bacteria at the concentration corresponding to maximum dose allowed for spraying in vineyards included Cantus® (boscalid) and Prolectus® (fenpyrazamine), mainly used to control powdery mildew and/or secondary rots. The former is a carboxamide fungicide inhibitor of succinate dehydrogenase, the latter an amino-pyrazolone that inhibits ergosterol biosynthesis. Ridomil Gold ® is used mainly against downy mildews in vineyards and contains metalaxyl-M, which acts on the polymerase complex of rRNA synthesis of fungi. To the best of author’s knowledge, this is the first study on the effects of these three fungicides on bacteria. Previous investigations into the impact of boscalid and metalaxyl-M on bacteria concerned experiments with treated soil (Ahmed El-Imam and Machido 2012; Wang et al. 2020).

This work clearly demonstrates the effects of fungicides on grapevine bacteria, through in vitro experimentation. The effective impact of these molecules on bacterial microbiota in the field has yet to be evaluated. This result could be obtained with field trials by analyzing culturable and unculturable microbial communities of the grapevine.

## Conclusion

This study demonstrates that fungicides designed for protecting grapevines from the most important fungal pathogens have different effects on non-target bacteria. Inter- and intra-species sensitivity of bacteria, including those with PGP traits and antifungal activity, observed by screening many genera/OTU with several chemical classes of fungicides, is a new finding. The potential negative impact of fungicidal treatments on natural bacterial populations of grapevines has been clearly highlighted by the observation that most of the isolates involved are beneficial to plant growth. The evidence that fungicides that target the same fungal pathogen or disease have different inhibitory effects on these isolates paves the way for a discussion about the use of molecules that have less impact on the bacterial microbiota. For example, carboxamide or strobilurin fungicides, in particular trifloxystrobin, may be preferable to triazolic fungicides in controlling powdery mildew. Similarly, spray applications of boscalid or fenpyrazamine against secondary rots may be advisable as well as metalaxyl-M for controlling downy mildews. This practice may increase the positive effect of either autochthonous PGP bacteria or exogenous biocontrol and biofertilizer agents. The use of selected exogenous PGP bacteria, as alternative strategies to counter phytopathogenic fungi and improve plant nutrient assimilation, is becoming increasingly widespread. In particular, appropriate experiments should be carried out in the field to assess the antagonistic activity of these bacteria against obligate phytopathogens, such as powdery and downy mildew, against which fungicide treatments are necessary. In this context, investigations into the compatibility of a certain fungicide with selected bacterial biocontrol agents must be encouraged. Since the impact of fungicides on natural bacterial populations could favour the selection of resistant strains, it appears clear that any loss of microbial biodiversity due to fungicidal treatments should be avoided. Finally, the understanding of the mechanisms of action of these molecules on non-target microorganisms is a priority in order to design new eco-friendly pesticides.

## Electronic supplementary material

Below is the link to the electronic supplementary material.

Supplementary Material 1

## Data Availability

All data generated or analysed during this study are included in this published article (and its supplementary information files).
