# Supplementary material for: Fungicide sensitivity of grapevine bacteria with plant growth-promoting traits and antagonistic activity as non-target microorganisms
Source: World J Microbiol Biotechnol. 2023 Mar 17;39(5):121. doi: 10.1007/s11274-023-03569-5 (PMC10020324; doi:10.1007/s11274-023-03569-5)

**Supplementary materials**

Andreolli et al. “ Fungicide sensitivity of grapevine bacteria with plant growth-promoting traits and antagonistic activity as non-target microorganisms”

**Table S1.** List of 48 bacteria isolated from grapevines in this study.

|  |  |  |  |
| --- | --- | --- | --- |
| **Isolate** | **Organ** | **Habitat** | **Location** |
|  |  |  |  |
| VAe | trunk | endophytic | Verona |
| V3Be | trunk | endophytic | Verona |
| V3Ae | trunk | endophytic | Verona |
| V7e | petiole | endophytic | Verona |
| V5B | leaf | epiphytic | Verona |
| V5G | leaf | epiphytic | Verona |
| V81 | leaf | epiphytic | Verona |
| V82 | leaf | epiphytic | Verona |
| V12e | branch | endophytic | Verona |
| V111 | branch | epiphytic | Verona |
| S1E | rhizosfere | rhizopheric | Verona |
| S1B | rhizosfere | rhizopheric | Verona |
| S1D | rhizosfere | rhizopheric | Verona |
| S23 | rhizosfere | rhizopheric | Verona |
| S1A | rhizosfere | rhizopheric | Verona |
| S25 | rhizosfere | rhizopheric | Verona |
| V13B | leaf | epiphytic | San Giovanni Lupatoto |
| V13C | leaf | epiphytic | San Giovanni Lupatoto |
| V13E | leaf | epiphytic | San Giovanni Lupatoto |
| V13F | leaf | epiphytic | San Giovanni Lupatoto |
| V13M | leaf | epiphytic | San Giovanni Lupatoto |
| VV13A | leaf | epiphytic | San Giovanni Lupatoto |
| V101 | leaf | epiphytic | San Giovanni Lupatoto |
| PT1e | leaf | endophytic | Trambileno |
| PT2e | leaf | endophytic | Trambileno |
| PT2A | leaf | epiphytic | Trambileno |
| PT13 | leaf | epiphytic | Trambileno |
| VT2 | leaf | epiphytic | Trambileno |
| PT14 | leaf | epiphytic | Trambileno |
| PT2D | leaf | epiphytic | Trambileno |
| VT3 | leaf | epiphytic | Trambileno |
| VT1 | leaf | epiphytic | Trambileno |
| PT11 | leaf | epiphytic | Trambileno |
| LG2 | leaf | epiphytic | Avio |
| LG1 | leaf | epiphytic | Avio |
| LG5B | leaf | epiphytic | Avio |
| LG6 | leaf | epiphytic | Avio |
| LG3 | leaf | epiphytic | Avio |
| LG4M | leaf | epiphytic | Avio |
| LG4T | leaf | epiphytic | Avio |
| LG5A | leaf | epiphytic | Avio |
| V12e | leaf | endophytic | Oppeano |
| VO22 | leaf | epiphytic | Oppeano |
| VO33 | leaf | epiphytic | Oppeano |
| VO21 | leaf | epiphytic | Oppeano |
| VO32 | leaf | epiphytic | Oppeano |
| VO1 | grape berry | epiphytic | Oppeano |
| ITAVB | leaf | epiphytic | Lonigo |
| ITAVA | leaf | epiphytic | Lonigo |
| ITAVF | leaf | epiphytic | Lonigo |
| ITAVE | leaf | epiphytic | Lonigo |
| ITAVG | leaf | epiphytic | Lonigo |
| FM2 | grape berry | epiphytic | Breganze |
| FM5 | grape berry | epiphytic | Breganze |
| FM15 | grape berry | epiphytic | Breganze |
| FM1 | grape berry | epiphytic | Breganze |
| FM6 | grape berry | epiphytic | Breganze |
| G2 | grape berry | epiphytic | Cazzano di Tramigna |
| G5 | grape berry | epiphytic | Cazzano di Tramigna |

**Table S2.** General properties of commercial fungicides used in this study.

|  |  |  |  |  |  |  |  |  |  |  |
| --- | --- | --- | --- | --- | --- | --- | --- | --- | --- | --- |
| **Trade name** | **Company** | **Formulation** | **Active ingredient (%)** | **MOA code** | **Group name** | **Chemical group** | **FRAC**  **code** | **Propriety** | **Target disease** | **Maximum individual dose^1^** |
|  |  |  |  |  |  |  |  |  |  |  |
| Dedalus®  SE | Adama Italia | emulsifiable concentrate | tebuconazole  (43.1) | G1 | demethylation inhibitor | triazole | 3 | systemic | powdery mildew | 230 mL/100 L |
| Lidal® | Corteva agriscience | emulsifiable concentrate | tetraconazole  (3.85) | G1 | demethylation inhibitor | triazole | 3 | systemic | powdery mildew | 375 mL/100 L |
| Topas®  10 EC | Syngenta Italia | emulsifiable concentrate | penconazole  (10.1) | G1 | demethylation inhibitor | triazole | 3 | systemic | powdery mildew  black rot | 30 mL/100 L |
| Ridomil®  Gold SL | Syngenta Italia | soluble liquid | metalaxyl-M  (43.9) | A1 | RNA polimerase I | acylalanine | 4 | systemic | downy mildew | 23 mL/100 L |
| Cantus® | BASF Italia | water-soluble granules | boscalid  (50.0) | C2 | succinate-dehydrogenase inhibitor | pyridine-carboxamide | 7 | foliar, translaminar | powdery mildew  secondary rots | 120 g/100 L |
| Switch® | Syngenta Italia | water-soluble granules | cyprodinil  (37.5) | D | methionine biosynthesis | anilino-pyrimidine | 9 | foliar, partially systemic | secondary rots | 80 g/100 L |
|  |  |  | fludioxonil  (25.0) | E | MAP protein kinase in osmotic signal transduction | phenylpyrrole | 12 |  |  |  |
| Prolectus® 50 WG | Sumitomo chemical Italia | water-soluble granules | fenpyrazamine  (50.0) | G3 | keto reductase inhibitor | aminopyrazolinone | 17 | translaminar | secondary rots | 100 g/100 L |
| Tucana®  25 EC | BASF Italia | emulsifiable concentrate | pyraclostrobin  (23.6) | C3 | quinone oustide inhibitor | methoxy-carbamate | 11 | foliar, translaminar | powdery mildew | 40 mL/100 L |
| Flint® | Bayer Italia | water-soluble granules | trifloxystrobin  (50.0) | C3 | quinone oustide inhibitor | oximino-acetate | 11 | foliar, translaminar | powdery mildew | 25 g/100 L |
| Carson®  45 WG | Adama Italia | water-soluble granules | cymoxanil  (45.0) | U | unknown | cyanoacetamide-oxime | 27 | translaminar | downy mildew | 135 g/100 L |
| Folpan®  80 WDG | Adama Italia | water-soluble granules | folpet  (80.0) | M | multi-site contact activity | phthalimide | M04 | foliar | downy mildew | 200 g/100 L |

^1^ maximum individual dose allowed for each application in vineyards reported in the approved label

**Figure S1.** Group of isolates tolerant to all fungicides and 15 groups of sensitive strains (S1A-S7) according to sensitive pattern of isolates to each fungicides (Dedalus®, D; Lidal®, L; Topas®, T; Switch®, S; Tucana®, Tu; Carson®, C; Folpan®, F).


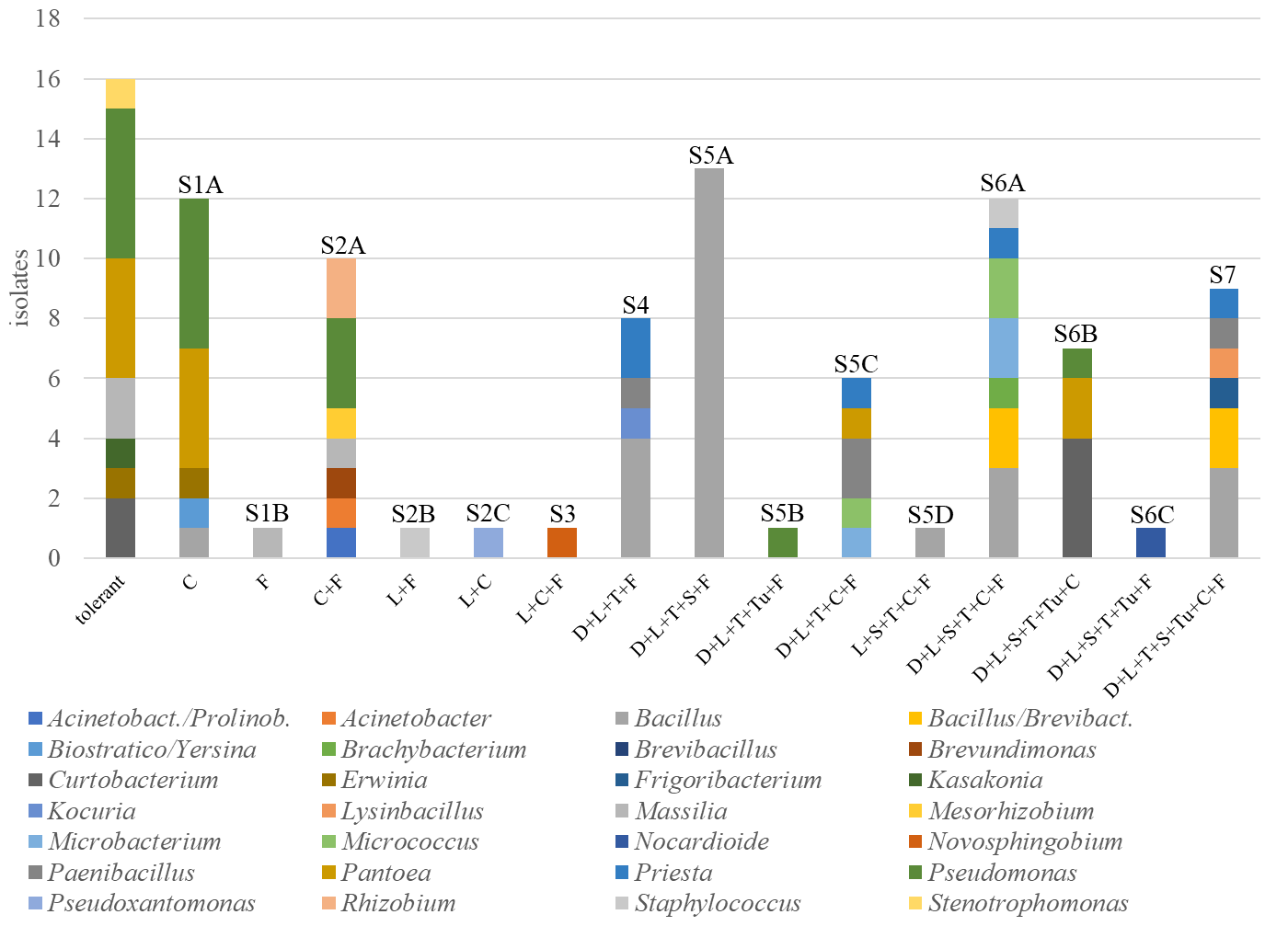

Supplement: Supplementary file 1 — Supplementary Material 1 [file 11274_2023_3569_MOESM1_ESM.docx]
